# Impact of Obesity on Serum Concentrations of Vancomycin Administered as Continuous Infusion and on Clinical Outcomes in Critically Ill Patients—A Retrospective Observational Study

**DOI:** 10.3390/antibiotics14090895

**Published:** 2025-09-04

**Authors:** Stefanie Nothofer, Rico Angeli, Manfred Weiss, Christian Dumps, Felix Berger, Josephin Eckert, Felix Girrbach, Nadin Scheidt, Susan Menzel, Mirko Lange, Hermann Wrigge, Philipp Simon

**Affiliations:** 1 Anaesthesiology and Operative Intensive Care, Faculty of Medicine, University of Augsburg, 86156 Augsburg, Germany; stefanie.nothofer@uni-a.de (S.N.); rico.angeli@uk-augsburg.de (R.A.); manfred.weiss@uk-augsburg.de (M.W.); christian.dumps@uk-augsburg.de (C.D.); felix.berger@uk-augsburg.de (F.B.); felix.girrbach@uk-augsburg.de (F.G.); 2 Department of Anaesthesiology and Intensive Care, University of Leipzig Medical Center, 04103 Leipzig, Germany; josephin.eckert@sana.de (J.E.); hausarztleutzsch@web.de (N.S.); susan.menzel@helios-gesundheit.de (S.M.); m.lange@deutschefachpflege.de (M.L.); 3 Department of Anaesthesiology, Intensive Care, Emergency Medicine and Pain Therapy, Bergmannstrost Hospital Halle, 06112 Halle, Germany; hermann.wrigge@bergmannstrost.de

**Keywords:** critically ill, continuous infusion, intensive care unit, obesity, serum concentrations, therapeutic drug monitoring, vancomycin

## Abstract

**Background/Objectives:** Vancomycin is a commonly used antibiotic in critically ill patients with severe methicillin-resistant *Staphylococcus aureus* infections. Due to its narrow therapeutic window, under- or overdosing is likely to result in adverse effects, especially in patients with conditions associated with altered pharmacokinetics such as obesity. The objective of this study was to investigate the impact of obesity on serum concentrations of vancomycin in critically ill patients receiving intravenous vancomycin by continuous infusion based on ideal body weight (IBW). **Methods**: This retrospective observational study performed at the University Hospital of Leipzig, Germany, included all patients admitted to the intensive care unit (ICU) between January 2009 and December 2015 who received guideline-based vancomycin therapy based on IBW. Serum concentrations were obtained through routinely performed therapeutic drug monitoring (TDM). **Results**: A total of 1066 patients with a median age of 62 years were included in this study. The median (25%; 75% quantile) vancomycin treatment duration was 4 (2; 7) days and the median time to reach target concentrations of 20–25 mg L^−1^ was 3 (2; 4) days without a significant difference between BMI groups. Overall, only 25.9% of patients were in the therapeutic range of 20–25 mg L^−1^ in the entire treatment interval. 47.8% of vancomycin concentrations obtained from TDM were below the desired target range with no differences between the BMI groups (*p* = 0.077). 26.3% of measurements exceeded the target range, with a significant increase in the morbidly obese group (*p* < 0.001). A higher BMI was associated with an increased ICU, in-hospital, 28- and 90-day mortality in morbidly obese patients (*p* < 0.05). Age, BMI and high SAPS-II and SOFA scores were significant predictors of an increased risk of death. **Conclusions**: Our preliminary findings suggest that IBW-based dosing may help reduce the risk of supratherapeutic concentrations in morbidly obese patients. The high rates of sub- and supratherapeutic vancomycin serum concentrations across all patients highlight the need for close TDM and dose adjustments, particularly in morbidly obese patients with the highest rates of supratherapeutic vancomycin serum concentrations and of RRT.

## 1. Introduction

Effective and precise management of antibiotic therapy is essential to optimize therapeutic efficacy while minimizing adverse effects. For antimicrobial agents with a narrow therapeutic window such as vancomycin, therapeutic drug monitoring (TDM) is routinely performed. Vancomycin is a hydrophilic glycopeptide antibiotic with potent activity against Gram-positive bacteria and is regarded as the agent of choice for the treatment of severe infections caused by methicillin-resistant *Staphylococcus aureus* (MRSA) and coagulase-negative *Staphylococci*. Despite more than 60 years of clinical use, controversies persist regarding optimal dosing regimens, methods of administration, and TDM targets, particularly in patients with potentially altered pharmacokinetics (PK), which is likely the case in obese or critically ill patients.

Obesity represents a significant risk factor for the development and treatment of severe infections [1,2]. Patients with morbid obesity exhibit altered and highly variable PK, which can lead to unpredictable and potentially inadequate therapeutic outcomes [3]. Factors contributing to these changes include increased cardiac output, renal and hepatic blood flow, reduced tissue perfusion, a higher body fat percentage, and greater total body water, resulting in an expanded volume of distribution (V_d_) [4,5]. In critically ill patients, the V_d_ may be further increased due to capillary leak and third-spacing, while renal and hepatic clearance are frequently impaired as a consequence of acute kidney injury (AKI) or organ failure [6,7,8]. Vancomycin administration via continuous infusion (CI) has been promoted as an alternative to intermittent infusion (II), offering potential advantages such as earlier attainment of the therapeutic target range [9], reduced variability in serum concentrations and simplified TDM with less dependence on sampling time [10], as well as a lower risk of AKI in critically ill patients [9,11,12]. It remains unclear whether these advantages also apply to obese patients.

For critically ill patients with normal kidney function, the American Society of Health Pharmacists guideline recommends a vancomycin loading dose of 15–20 mg kg^−1^ based on total body weight (TBW), not exceeding 3 g, followed by daily maintenance doses of 30–40 mg kg^−1^ TBW administered over a period of 8–12 h as CI [13]. In contrast to the 2009 guideline, the updated 2020 version also provides specific recommendations for dosing in obese patients, suggesting loading doses of 20–25 mg kg^−1^ TBW, not exceeding 2 g [13,14]. For critically ill patients receiving vancomycin as CI, steady-state concentrations should be maintained between 20 and 25 mg L^−1^ [9]. However, the nonlinear relationship between vancomycin V_d_ and TBW creates a risk of supratherapeutic concentrations and of toxicity-related complications such as AKI in obese patients, in part due to the calculation of maintenance doses based on the TBW [15,16,17,18]. To mitigate this, dosing based on the ideal rather than the total body weight has been proposed [19].

Given the complex and sometimes contradictory effects of obesity on vancomycin drug disposition, critical evaluation of current dosing regimens is crucial to ensure effective treatment of those patients. The objective of this retrospective observational study was to investigate the impact of obesity on the serum concentrations of vancomycin dosed based on IBW and administered as CI in critically ill patients, as well as its association with adverse events such as AKI, renal replacement therapy (RRT) and short-term outcomes.

## 2. Results

### 2.1. Study Population

**Figure 1:**
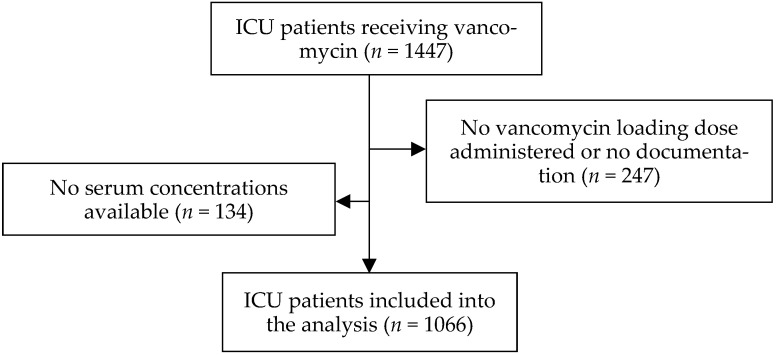
PRISMA flowchart of the study population. ICU: intensive care unit; PRISMA: Preferred Reporting Item for Systematic reviews and Meta-Analyses [20].

After exclusion of 381 patients for whom TDM was not performed, who did not receive a vancomycin loading dose or without reliable documentation of such, a total of 1066 patients (65.5% male) with a median (25%, 75% quantile) age of 62 (51; 73) years and a median BMI of 26.1 (23.6; 29.4) kg m^−2^ were included in the final analysis (see Figure 1). Patient characteristics are displayed in Table 1. The median SOFA and SAPS-II score as well as the necessity for mechanical ventilation on the day of admission did not differ between the BMI groups. However, significantly more severely and morbidly obese patients received RRT even before initiation of vancomycin therapy (*p* < 0.001).

### 2.2. Vancomycin Treatment Course and TDM Results

**Figure 2:**
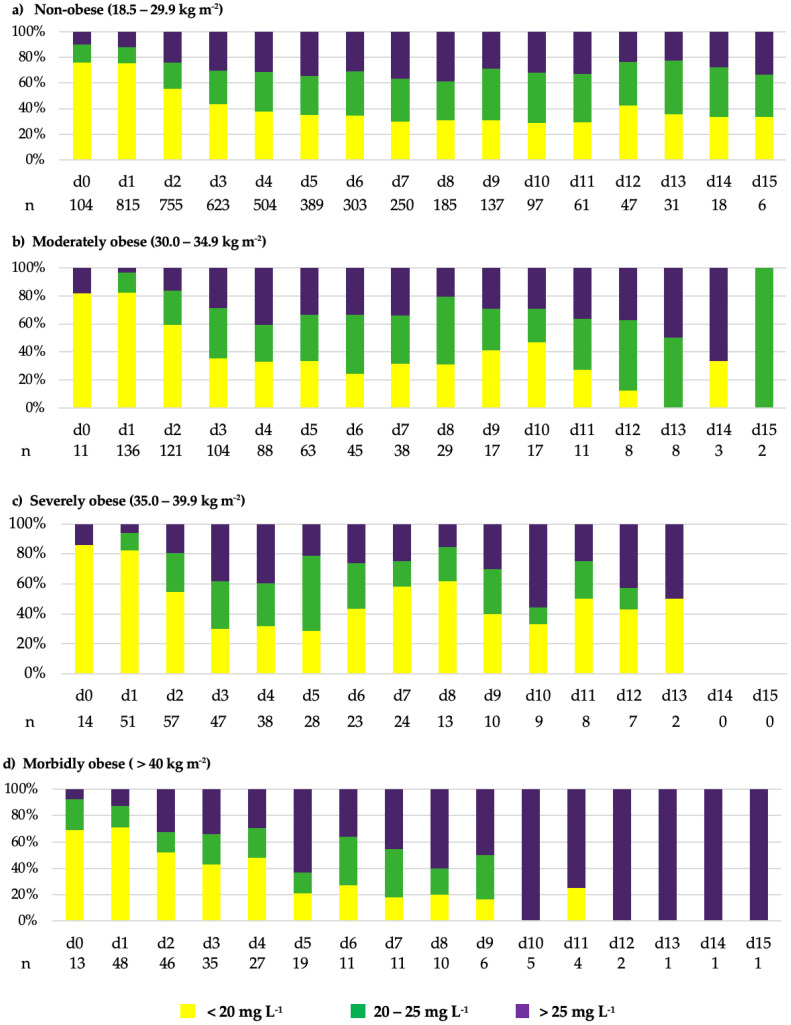
Vancomycin steady-state concentrations from day 0 to 15 stratified by BMI groups. Detailed graphical illustration of the proportions of measured vancomycin steady-state concentrations below, above and in the target range over the entire treatment period stratified by BMI groups, with color-coded classification into target range (20–25 mg L^−1^, green), underdosing (<20 mg L^−1^, yellow), and overdosing (>25 mg L^−1^, purple) separated into non-obese (**a**), moderately obese (**b**), severely obese (**c**) and morbidly obese patients (**d**).

The vancomycin treatment course as well as the TDM results are displayed in Table 2. Serum concentrations after initiation of vancomycin therapy (day 0) were available for 853 (80.0%) of 1066 patients on day 1. In total, 4699 vancomycin steady-state concentrations were measured. The number of vancomycin serum concentration measurements per patient did not differ between the BMI groups. Overall, median (25%; 75% quantile) vancomycin treatment duration was 4 (2; 7) days and the median time from the first vancomycin administration to reaching the targeted steady-state concentration of 20–25 mg L^−1^ was 3 (2; 4) days. Overall, 25.9% of measured vancomycin concentrations were within the target range with no significant difference between the BMI groups. Furthermore, 79% and 41% of patients did not reach the targeted vancomycin concentrations on day 1 and day 3, respectively. Subtherapeutic vancomycin concentrations < 20 mg L^−1^ were measured in 83.2% of patients at least once between day 1 and 15. Overall, 47.8% of vancomycin concentrations obtained from TDM were below the desired target range without statistically significant differences between the groups (*p* = 0.077). No significant difference was observed in the number of days below the target range in relation to the total therapy days between the groups (*p* = 0.757). Supratherapeutic vancomycin concentrations > 25 mg L^−1^ were measured at least once in 303 (28.4%) patients. Overall, 26.3% of TDM measurements were above the targeted range, with a significant increase in the morbidly obese patient group (*p* < 0.001). However, there was no significant difference when days of supratherapeutic concentrations were compared to the total vancomycin therapy days (*p* = 0.529). The number of measured vancomycin steady-state concentrations below (<20 mg L^−1^), above (>25 mg L^−1^), and within the target range (20–25 mg L^−1^) over the entire treatment period, as well as their proportions relative to each other, are illustrated stratified by BMI group in Figure 2. Note that in the morbidly obese group, concentrations measured between days 13 and 15 were repeatedly obtained from the same patient, which may have artificially increased the proportion of supratherapeutic concentrations in this group.

### 2.3. Bacterial Species

**Figure 3:**
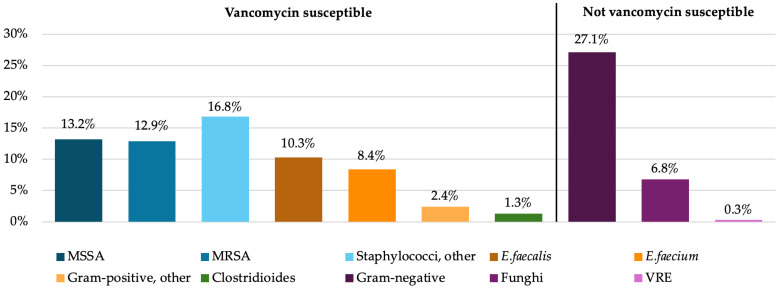
Detected microorganisms in microbial samples. MSSA: methicillin-susceptible *Staphylococcus aureus*; MRSA: methicillin-resistant *Staphylococcus aureus*: *E. faecalis*: *Enterococcus faecalis*; *E. faecium: Enterococcus faecium*; VRE: vancomycin-resistant *Enterococci*.

Microbiological testing was performed before initiation of therapy, either by obtaining samples from the organ system suspected to be the primary source of infection and/or via blood culture incubation. The most frequently detected pathogens were Gram-positive species (72.9%) followed by Gram-negative species (27.1%). Methicillin-resistant *staphylococcus aureus* was detected in 12.9% of patients (see Figure 3). Patients with infections from pathogens not susceptible to vancomycin received additional antibiotic therapy with appropriate agents. A detailed overview of the detected pathogens and sample locations can be found in Supplementary Table S1.

### 2.4. Disease Severity and Outcome Measures

**Figure 4:**
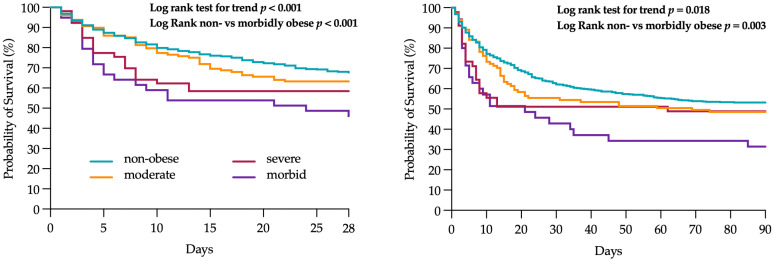
28-day mortality (**left**) and 90-day mortality (**right**).

The median SAPS-II and SOFA scores during the vancomycin treatment interval were higher in patient groups with a higher BMI with a significant difference between the non-obese and the severely obese patients (see Table 3). Overall, 81.1% of patients were mechanically ventilated at any point during the observation period with a median duration of 3 (1; 6) days. 35.2% of patients required renal replacement therapy (RRT) for a median of 3 to 4 days at any point during the vancomycin treatment interval with a significant increase among severely obese (19.0%) and morbidly obese (15.9%) compared to non-obese patients (*p* < 0.001). Overall, 32.1% of patients died in the ICU and 38.0% during the hospital stay with a significant increase towards the morbidly obese group. The same pattern was observed for the 28-day mortality (see Figure 4).

### 2.5. Impact of RRT and BMI on Vancomycin Serum Concentrations

As RRT is a well-recognized determinant of vancomycin therapy management and showed significant differences across BMI groups (see Table 3), a multivariate analysis of variance (MANOVA) was conducted to assess the effects of BMI classification, the necessity for RRT and their interaction on vancomycin exposure (see Supplementary Tables S2–S4). The intercept was highly significant, indicating that the overall multivariate means of the dependent variables, i.e., BMI and RRT, differed significantly from zero (Pillai’s Trace = 0.622, *p* < 0.001). Neither the main effects of BMI group (Pillai’s Trace = 0.018, *p* = 0.811) and RRT (Pillai’s Trace = 0.011, *p* = 0.291) nor the interaction between BMI group and RRT were significant (Pillai’s Trace = 0.023, *p* = 0.608). RRT did not significantly affect any dependent variable regarding vancomycin therapy when comparing patient groups with and without controlling for BMI.

### 2.6. 28- and 90-Day Mortality Stratified by BMI Groups

Survival analysis was performed with curves and differences in 28- and 90-day survival in the BMI groups were tested with the Log-Rank test for trend. Time to event was defined as the time from the first day of vancomycin administration to 28 days thereafter. As depicted in Figure 4, Kaplan–Meier survival analysis showed a significant difference in the survival curves among the four groups regarding the 28-day mortality overall (Log-rank test for trend *p* < 0.001) and by pairwise comparison between the non-obese and morbidly obese patients (Log-rank test *p* < 0.001, see Figure 4). A comparable result was observed for the 90-day mortality (Log rank for trend *p* = 0.018), with a significantly higher survival probability in the non- vs. morbidly obese group (*p* = 0.003).

### 2.7. Post Hoc Analysis of Outcomes by Vancomycin Concentrations

A post hoc analysis was performed to evaluate clinical outcomes stratified by the occurrence of supratherapeutic vancomycin serum concentrations at any point during the treatment interval (see Table 4). Patients in whom supratherapeutic serum concentrations were recorded at least once had significantly higher ICU, in-hospital, and 90-day mortality rates compared with those who never reached supratherapeutic concentrations. Similarly, the highest recorded SOFA and SAPS-II scores, as well as length of stay in the ICU and in the hospital were markedly increased in patients who had at least one vancomycin serum concentration exceeding 25 mg L^−1^.

## 3. Discussion

This study represents the largest cohort investigating the potential impact of obesity on vancomycin serum concentrations as well as disease severity and associated outcome measures. The primary finding of this retrospective observational study is that obesity and its varying degrees do not significantly affect the likelihood of achieving the targeted steady-state vancomycin concentrations in critically ill patients receiving vancomycin as continuous infusion. Secondary analyses revealed that the ICU, in-hospital, 28-day and 90-day mortality rates were significantly higher among patients with morbid obesity and in patients in whom vancomycin concentrations exceeded the therapeutic range of 20–25 mg L^−1^ at least once during the treatment interval.

In this study cohort, vancomycin therapy was managed through administration of an initial bolus followed by daily doses based on IBW as continuous infusion guided by daily measurement of steady-state concentrations. Although this approach was used for all included patients, only 25.9% of patients achieved therapeutic concentrations of 20–25 mg L^−1^ in the entire treatment interval. According to our results, obesity does not significantly influence the achievement of targeted steady-state vancomycin concentrations. A possible explanation could be the hydrophilic properties of vancomycin, which limit its ability to freely diffuse into all body tissues, thereby preventing the V_d_ from increasing linearly with TBW in obese patients [6]. Previous studies demonstrated that obesity-specific models regarding the PK of vancomycin could not provide an advantage over general-purpose models based on normal-weight patients [8]. This consideration along with our findings suggests that vancomycin dosing in critically ill, obese patients may be more appropriate when based on ideal rather than total body weight, especially given the substantial risk of adverse effects associated with increasing doses. This concept is supported by a recent study that identified IBW as a better predictor of reaching vancomycin steady state concentrations of 15–20 mg m L^−1^ than TBW [19]. The lack of association between obesity and supratherapeutic vancomycin concentrations stands in contrast to previous studies that identified obesity as a risk factor for vancomycin overdose [21,22], supporting the use of IBW as a size metric for dose calculation and the advantage of continuous rather than intermittent infusion. TBW-based dosing naturally leads to higher doses being administered to patients with above normal body weight. All patients in this investigation received similar loading and cumulative doses irrespective of their weight. Despite receiving similar IBW-based doses, serum concentrations of morbidly obese patients were significantly more often in the supratherapeutic range compared to the lower BMI groups. Thus, TBW-based dosing would have led to an even higher rate of overdosing, at least in the morbidly obese patients. However, this may reflect a statistical bias resulting from the lower number of cases in the higher BMI groups, the progressively declining number of TDM measurements over time, or the relatively short median treatment duration of 3 to 4 days, all of which could limit the robustness of the findings in these subgroups. Consistent with this, patients with supratherapeutic vancomycin concentrations at any point during treatment demonstrated significantly higher 90-day, ICU, and in-hospital mortality rates, greater disease severity as reflected by higher SAPS II and SOFA scores and longer stays in both the ICU and the hospital. Overdosing becomes especially relevant when considering that nephrotoxicity is one of the most prevalent adverse effects of vancomycin and has previously been shown to be increased in patients with obesity [15,16,23,24]. In our study, the incidence of AKI and its stages according to the AKIN classification after onset of vancomycin treatment did not differ between the BMI groups, yet significantly more patients with severe and morbid obesity required renal replacement therapy. However, the higher percentage of morbidly obese patients requiring RRT compared to the other patient groups was already present at baseline before initiation of vancomycin therapy. As kidney function and RRT are well-known determinants of vancomycin therapy management, we examined the effects of BMI group, RRT, and their interaction on the probability of achieving sub-, target-range, or supratherapeutic concentrations. Neither BMI, RRT, nor their interaction showed a significant impact. Additionally, we found an increased ICU-, in-hospital- as well as 28-day and 90-day mortality in the higher BMI groups. This association with vancomycin therapy must be interpreted with caution because obese patients did not receive higher vancomycin doses overall. Furthermore, numerous pathophysiological changes in critically ill patients with sepsis and septic shock as well as individual comorbidities and the underlying disease itself make it difficult to directly link vancomycin administration to the need for RRT or even mortality rates [25,26].

As far as outcome measures are concerned, the relationship between BMI and mortality has been shown to be more complex than anticipated. While large-scale meta-analyses have demonstrated increased all-cause mortality in obese patients [27,28], the ‘obesity paradox’ refers to the phenomenon that obesity is associated with survival benefits regarding short-term outcomes from critical illness [27,28,29,30,31]. In our study, survival analysis by Kaplan–Meier curves demonstrated that a higher BMI was indeed associated with an increased 28- and 90-day mortality. However, due to the multiple factors that come into play when assessing mortality in critically ill patients, no direct causal relationship can be established, but merely an association. While we did assess some of these factors, including daily SAPS-II and SOFA scores as well as necessity of RRT and mechanical ventilation, we ultimately excluded the co-variates after baseline from the multivariate analysis, as they could have been impacted by the vancomycin treatment itself.

In contrast to the 2009 guideline, the recently updated guideline on vancomycin therapy by Rybak et al. [13] no longer recommends trough only monitoring, as the trough represents a single exposure point at the end of the dosing interval, translating into one specific minimum daily value of the area under the curve (AUC). Instead, the PK/PD index of AUC over 24 h (AUC_24h_) to minimum inhibitory concentration (MIC) ratio (AUC_24h_/MIC) with a target range of 400–600 h × mg L^−1^ based on a MIC of 1 mg L^−1^ is now proposed for dose guiding, as the AUC_24h_ largely represents the average concentration during the entire 24 h period. The recommended range is largely based on studies demonstrating favorable clinical outcomes while minimizing the risk of over-exposure and adverse events such as AKI [15,32,33]. It should be noted that the PK/PD target for CI has not been validated as most PK data supporting an AUC_24h_/MIC ratio of > 400 as the best predictor of a favorable clinical outcome are derived from patients receiving vancomycin via intermittent infusion.

There are some limitations to this study. The data of this observational analysis date back to the years 2009 to 2016. Therefore, TDM and any dosing adjustments were based on measurements of steady-state concentrations instead of the AUC_24_/MIC. Furthermore, we employed a target range of 20–25 mg L^−1^ as recommended for critically ill patients with severe MRSA infections. It could be argued that for critically ill patients with infections caused by other Gram-positive bacteria, a lower target range of 15–20 mg L^−1^ might have been adequate; however, this remains to be investigated, especially for CI. Given the retrospective design of this study, only statistical associations can be observed, but no causal inferences can be made. Additionally, we refrained from including a multivariate analysis on the primary endpoint of reaching supratherapeutic concentrations above 25 mg L^−1^ during the treatment interval. While we initially performed such an analysis, it yielded no significant results, meaning neither subtherapeutic (*p* = 0.199) nor supratherapeutic vancomycin concentrations (*p* = 0.581) had a significant impact on the outcome. As most patients showed sub- or supratherapeutic concentrations at any point during treatment, the discriminatory power of such an analysis was very limited anyway, and any cut-off would have been arbitrary. It should also be acknowledged that additional confounders, most notably renal function and the need for RRT, are likely to play an important role in this context but were not examined. The morbidly obese group exhibited higher rates of RRT and mortality. Due to the retrospective study design, we were unable to directly compare outcomes within BMI groups against expected risk factors for supratherapeutic vancomycin serum concentrations, such as RRT or creatinine clearance. Consequently, it is difficult to discern whether supratherapeutic vancomycin concentrations were attributable to renal dysfunction, as indicated by reduced creatinine clearance and/or RRT, or to BMI itself. Finally, we did not assess concentration-outcome relationships within BMI groups as the number of patients with severe and morbid obesity would have been too small to investigate vancomycin-related outcomes such as mortality, AKI or RRT. Thus, this limits the interpretation of our dosing recommendations to a certain extent, as it remains unclear whether the higher rate of RRT and the higher mortality in the morbidly obese group were caused by supratherapeutic vancomycin serum concentrations. Accordingly, our results should be regarded as preliminary and hypotheses-generating, requiring confirmation in prospective, controlled studies with BMI-specific outcome validation.

## 4. Materials and Methods

### 4.1. Study Design and Patient Population

This retrospective observational study was conducted at the University Hospital of Leipzig, Germany, between 2009 and 2016. Ethical approval was granted by the Ethics Committee of the University of Leipzig (No. 426/16-lk), and the study was registered in the German Clinical Trials Register (DRKS00011176). Adult ICU patients with a BMI > 18.5 kg m^−2^ who received guideline-based vancomycin therapy via CI based on IBW were included. Obesity was defined according to the definition by the World Health Organization (WHO): moderate (Class I; 30.0–34.9 kg m^−2^); severe (Class II; 35.0–39.9 kg m^−2^) and morbid (Class III; >40.0 kg m^−2^) [34]. Patients with any kind of deviation from guideline recommendations on vancomycin dosing, i.e., patients for whom no loading dose was administered or documented or for whom TDM was not performed, were excluded from the final analysis.

### 4.2. Vancomycin Dosing Regimen and TDM

A loading dose of 15–20 mg kg^−1^ based on IBW rounded to the nearest 250 mg increment was administered on the first treatment day (day 0). This was followed by a daily maintenance dose of 30–40 mg kg^−1^ based on IBW and on the measured serum steady state concentrations. Vancomycin was administered via continuous infusion in all patients. TDM was performed daily until the end of vancomycin therapy. If subtherapeutic vancomycin serum concentrations were measured, an additional dose of 15–20 mg kg^−1^ based on IBW was administered. In case of supratherapeutic concentrations, vancomycin therapy was discontinued for one day and resumed once concentrations obtained from TDM were within the target range.

### 4.3. Study Endpoints

The primary endpoint was defined as the incidence of supratherapeutic vancomycin serum steady state concentrations > 25 mg L^−1^; hence the total number of patients where supratherapeutic vancomycin steady state concentrations were measured at least once during the treatment period; based on target steady-state concentrations of 20–25 mg L^−1^ as recommended by guidelines [13]. Secondary endpoints included the incidence of subtherapeutic serum concentrations < 20 mg L^−1^, the time to reach the targeted steady-state concentrations, rate and stage of AKI based on the Acute Kidney Injury Network (AKIN) classification, necessity of RRT, length of stay in the ICU and the hospital as well as the ICU-, in-hospital, 28-day and 90-day mortality rate.

### 4.4. Data Collection

The following data were extracted from electronic patient records from day 0 (treatment initiation day) to day 15: baseline characteristics including age, sex, height, TBW, IBW, BMI according to the current WHO definition [35], date of hospital and ICU admission, date of hospital and ICU discharge, date of in-hospital death, start and end of vancomycin therapy, daily vancomycin dose and additional boluses (if administered) and daily vancomycin serum steady-state concentrations. To assess organ failure and associated complications, the Simplified Acute Physiology Score Version II (SAPS-II) and the Sequential Organ Failure Assessment (SOFA) Score, necessity and cumulative days of renal replacement therapy (RRT), necessity of mechanical ventilation and ventilation-free days, diuresis, volume balance and inflammation parameters were documented daily. Ideal body weight was calculated using the Broca Index.

### 4.5. Bioanalytical Assay

Vancomycin serum concentrations were quantified using a validated enzyme immunoassay (EIA) method, performed on a Cobas^®^ 8000 modular analyzer (Roche Diagnostics, Mannheim, Germany) as part of clinical routine. Blood samples were collected in lithium heparin tubes at steady state, corresponding to steady state concentrations at approximately five o’clock in the morning.

### 4.6. Statistical Analysis

All data were documented in a Microsoft Excel spreadsheet (Microsoft Corporation, Redmond, WA USA). Statistical analysis was performed with JASP (Version 0.19.3., The JASP Team, Amsterdam, The Netherlands). Categorial variables are displayed as absolute (relative) numbers, continuous variables as median (25%; 75% quantile) unless stated otherwise. Continuous variables were compared between groups with the Kruskal–Wallis-test. Categorial variables were compared between groups by the Chi-square test. Bonferroni correction was applied when necessary. A multivariate analysis of variance (MANOVA) was conducted to assess the impact of BMI group, necessity of RRT and their interactions on the serum steady-state concentrations. Pillai’s Trace was used to assess multivariate significance, as it is considered robust to violations of assumptions such as unequal covariance matrices. Kaplan–Meier curves were used to assess 28- and 90-day survival between the BMI groups. Statistical comparison of the survival curves was performed by the Log-rank test for trend. A *p*-value < 0.05 was defined as statistically significant.

## 5. Conclusions

Our preliminary findings suggest that IBW-based dosing may help reduce the risk of supratherapeutic concentrations in morbidly obese patients. The high rate of sub- and supratherapeutic vancomycin serum concentrations underlines the necessity for close TDM to keep patients within the target range, especially those with morbid obesity, who exhibited the highest rate of supratherapeutic vancomycin concentrations and of RRT. However, in light of the abovementioned limitations, prospective controlled trials are needed to further assess IBW-based vancomycin dosing in obese patients on outcomes such as acute kidney injury and mortality.

## Figures and Tables

**Table 1:** Baseline characteristics and disease severity on the day of admission stratified by BMI.

BMI (kg m^−2^)	Total	18.5–29.9	30.0–34.9	35.0–39.9	>40	*p*-Value
Patients	1066	829	135	54	44	
Sex, male	699 (65.6)	562 (67.5)	81 (60.0)	29 (52.7)	27 (61.4)	0.050
Age (years)	62 (51; 73)	61 (50; 73)	64 (56; 75)	63 (56; 71)	58 (50; 67)	0.031
TBW (kg)	80 (70; 90)	75 (65; 82)	95 (85; 100)	110 (100; 120)	138 (122; 156)	<0.001
IBW (kg)	72 (60; 80)	72 (63; 80)	70 (59; 80)	70 (54; 80)	69 (59; 75)	0.098
BMI (kg m^−2^)	26.1 (23.6; 29.4)	24.7 (22.9; 27.1)	31.6 (30.9; 33.1)	37.0 (35.5; 38.7)	46.7 (42.5; 53.4)	<0.001
SOFA score	6 (4; 10)	6 (4; 10)	6 (4; 10)	7 (5; 11)	6 (4; 12)	0.792
SAPS-II score	43 (28; 57)	43 (28; 57)	46 (31; 59)	49 (33; 63)	39 (28; 57)	0.143
Necessity of RRT	234 (22.0)	163 (19.7)	37 (26.6)	19 (32.8)	15 (34.9)	<0.001
Necessity of MV	790 (74.1)	608 (73.6)	97 (69.8)	47 (81.0)	38 (88.4)	0.061

Data are presented as absolute (relative frequencies) for categorial variables and as median (25%; 75% quantile) for continuous variables. BMI: Body Mass Index; IBW: ideal body weight; MV: mechanical ventilation; RRT: renal replacement therapy; SOFA: Sequential Organ Failure Assessment, SAPS-II: Simplified Acute Physiology Score Version II, TBW: total body weight.

**Table 2:** Vancomycin treatment course stratified by BMI.

BMI (kg m^−2^)	Total (*n* = 1066)	18.5–29.9 (*n* = 829)	30.0–34.9 (*n* = 135)	35.0–39.9 (*n* = 54)	>40 (*n* = 44)	*p*-Value
Vancomycin therapy (days)	4 (2; 7)	4 (2; 7)	4 (3; 7)	4 (3; 8)	3 (2; 6)	0.723
No. of TDM measurements obtained from each patient	4 (2; 6)	4 (2; 6)	4 (2; 6)	4 (2; 7)	3 (2; 5)	0.446
TDM measurements (cum.) *	4699	3633	627	260	179	
<20 mg L^−1^	2247 (47.8)	1760 (48.4)	297 (47.4)	121 (46.5)	69 (38.6)	0.073
20–25 mg L^−1^	1218 (25.9)	931 (25.6)	177 (28.2)	75 (28.9)	35 (19.6)	0.077
>25 mg L^−1^	1234 (26.3)	942 (25.9)	153 (24.4)	64 (24.6)	75 (41.9)	<0.001
Days to target concentrations	3 (2; 4)	3 (2; 4)	3 (2; 4)	3 (2; 4)	3 (1; 4)	0.835
Therapy days < 20 mg L^−1^	2 (1; 3)	2 (1; 3)	2 (1; 3)	1 (1; 3)	1 (1; 2)	0.159
% of therapy days	50.0 (25.0; 100)	50.0 (25.0; 100)	50.0 (25.0; 100)	50.0 (25.0; 75.0)	50.0 (17.0; 100)	0.757
Therapy days 20–25 mg L^−1^	1 (0; 2)	1 (0; 2)	1 (0; 2)	1 (0; 2)	1 (0; 1)	0.189
% of therapy days	16.7 (0.0; 37.5)	14.3 (0.0; 37.5)	25.0 (0.0; 43.7)	25.0 (0.0; 40.7)	35.7 (0.0; 33.3)	0.190
Therapy days > 25 mg L^−1^	1 (0; 2)	0 (0; 2)	0 (0; 2)	1 (0; 2)	0 (0; 3)	0.579
% of therapy days	9.1 (0.0; 40.0)	0.0 (0.0; 42.9)	0.0 (0.0; 33.3)	23.1 (0.0; 40.0)	0.0 (0.0; 50.0)	0.529
Loading dose (g)	1.0 (1.0; 1.0)	1.0 (1.0; 1.0)	1.0 (1.0; 1.0)	1.0 (1.0; 1.0)	1.0 (1.0; 1.0)	0.180
Vancomycin cum. dose (g)	8.0 (5.4; 12.5)	8.1 (5.4; 12.7)	7.6 (5.5; 11.8)	8.0 (5.9; 12.6)	8.8 (4.5; 12.9)	0.919
Vancomycin dose per day/IBW (mg kg^−1^)	31.1 (22.6; 42.3)	31.0 (22.7; 41.5)	30.3 (21.8; 42.8)	31.3 (21.9; 44.1)	36.4 (24.5; 50.3)	0.259

* cumulative TDM measurements over the entire vancomycin treatment interval. Data are presented as absolute (relative frequencies) for categorial and as median (25%; 75% quantile) for continuous variables. BMI: Body Mass Index; IBW: ideal body weight; No.: number; cum.: cumulative; TDM: therapeutic drug monitoring.

**Table 3:** Disease severity and outcomes during the vancomycin treatment interval stratified by BMI group.

BMI (kg m^−2^)	Total	18.5–29.9	30.0–34.9	35.0–39.9	>40	*p*-Value
Median SOFA *	7 (3; 11)	7 (3; 10)	6 (3; 10)	9 (4; 12)	10 (3; 12)	0.024
Highest SOFA *	9 (6; 13)	9 (6; 13)	9 (5; 13)	12 (5; 15)	12 (6; 14)	0.122
Median SAPS-II *	43 (31; 55)	43 (30; 55)	43 (33; 55)	50 (36; 64)	48 (33; 62)	0.035
Highest SAPS-II *	57 (40; 71)	48 (33; 62)	57 (44; 73)	61 (50; 81)	57 (42; 74)	0.066
Mechanical ventilation **	865 (81.1)	664 (80.4)	112 (80.6)	49 (84.5)	40 (93.0)	0.193
Ventilated days	3 (1; 6)	3 (1; 6)	4 (1; 6)	4 (3; 8)	4 (2; 7)	0.228
AKI (highest stage)						0.805
AKIN stage 1	256 (27.2)	196 (26.7)	35 (30.2)	13 (26.0)	12 (30.0)	
AKIN stage 2	172 (18.3)	134 (18.3)	23 (19.8)	8 (16.0)	7 (17.5)	
AKIN stage 3	207 (22.0)	156 (21.3)	26 (22.4)	16 (32.0)	9 (22.5)	
RRT **	375 (35.2)	269 (32.6)	50 (36.0)	31 (53.4)	25 (58.1)	<0.001
RRT days (cum.)	4 (2; 6)	4 (2; 6)	4 (2; 7)	4 (3; 6)	2 (2; 4)	0.187
Length of stay, ICU	22 (9; 37)	21 (9; 36)	23 (9, 42)	23 (11; 38)	17 (7; 50)	0.783
Length of stay, hospital	35 (22; 60)	34 (22; 60)	41 (21; 60)	34 (22; 60)	39 (14; 71)	0.940
ICU mortality	343 (32.1)	255 (30.8)	41 (29.5)	22 (37.9)	25 (58.1)	0.002
In-hospital mortality	408 (38.0)	305 (36.9)	52 (37.4)	23 (39.7)	25 (58.1)	0.047
28-day mortality	331 (34.3)	241 (32.4)	47 (36.7)	22 (41.5)	21 (53.8)	0.025
90-day mortality	418 (50.0)	314 (48.2)	56 (54.4)	23 (51.1)	25 (69.4)	0.068

* SOFA and SAPS-II score were documented on each vancomycin treatment day and the median as well as the highest documented value was taken. ** Necessity of mechanical ventilation or RRT at any point during the vancomycin treatment interval. Data are presented as absolute (relative frequencies) for categorial variables and as median (25%; 75% quantile) for continuous variables. AKIN: Acute Kidney Injury Network Classification; RRT: renal replacement therapy; BMI: Body Mass Index; SOFA: Sequential Organ Failure Assessment Score; SAPS-II: Simplified Acute Physiology Score Version 2; ICU: intensive care unit, cum.: cumulative.

**Table 4:** Impact of supratherapeutic vancomycin concentrations on clinical outcomes.

Supratherapeutic Vancomycin Concentrations at Any Point (>25 mg L^−1^)			*p*-Value
	No	Yes	
28-day mortality	160 (33.9)	172 (34.9)	0.746
90-day mortality	186 (44.7)	233 (55.3)	0.002
ICU mortality	153 (28.8)	190 (35.3)	0.022
In-hospital mortality	175 (33.0)	231 (42.9)	<0.001
	No	Yes	
Highest SOFA score	52 (37; 66)	61 (44; 74)	<0.001
Highest SAPS score	8 (5; 12)	11 (6;15)	<0.001
Length of stay, ICU	18 (6; 30)	25 (13; 44)	<0.001
Length of stay, hospital	31 (19; 55)	41 (24; 66)	<0.001

Data are presented as absolute (relative frequencies) for categorial variables and as median (25%; 75% quantile) for continuous variables. ICU: intensive care unit; SOFA: Sequential Organ Failure Assessment Score; SAPS: Simplified Acute Physiology Score Version II.

## Data Availability

The data presented in this study are available on request from the corresponding author.

## Supplementary Materials

The following supporting information can be downloaded at: https://www.mdpi.com/article/10.3390/antibiotics14090895/s1, Supplementary Table S1: Localization and bacterial species detected in the study population. Supplementary Table S2: Multivariate analysis of variance on the impact of BMI and RRT on vancomycin serum concentrations stratified by BMI group. Supplementary Table S3: Multivariate Tests. Results of the multivariate analysis of variance on the impact of BMI, RRT and their interaction on vancomycin serum concentrations.; Supplementary Table S4: Multivariate analysis. Tests of between-subject effects.

## Abbreviations

The following abbreviations are used in this manuscript:

PK	Pharmacokinetics
TDM	Therapeutic drug monitoring
CI	Continuous infusion
II	Intermittent infusion
MIC	Minimum inhibitory concentration
MRSA	Methicillin resistant *Staphylococcus aureus*
V_d_	Volume of distribution
AKI	Acute kidney injury
AKIN	Acute Kidney Injury Network Classification
TBW	Total body weight
SOFA	Sequential Organ Failure Assessment Score
SAPS	Simplified Acute Physiology Score, Version II
RRT	Renal replacement therapy
ICU	Intensive care unit
WHO	World Health Organization
AUC	Area under the concentration-time curve
IBW	Ideal body weight
g	gram
