# Supplementary material for: Impact of Obesity on Serum Concentrations of Vancomycin Administered as Continuous Infusion and on Clinical Outcomes in Critically Ill Patients—A Retrospective Observational Study
Source: Antibiotics (Basel). 2025 Sep 4;14(9):895. doi: 10.3390/antibiotics14090895 (PMC12466686; doi:10.3390/antibiotics14090895)
Supplement: Supplementary file 1 [file antibiotics-14-00895-s001.zip › antibiotics-3776708-supplementary.pdf]

Table S1. Localization and bacterial species detected in the study population

	Airways	Lung	Bloodstream	Bone/Spine	Cardiovascular	Catheter associated	Central nervous system	Gastrointestinal	intraabdominal	Thoracal	Urogenital	Wounds	others	Total
Staphylococcus aureus	98	21	20	44	2	10	23	7	3	11	5	6	3	253 (25.9)
**Staphylococci, other**	4	34	52	11	1	1	38	0	16	0	2	3	2	164 (16.8)
**Streptococci**	1	3	0	1	1	0	2	0	1	0	0	0	0	9 (0.9)
Enterococcus faecalis	3	5	5	16	11	0	29	0	14	0	15	2	1	101 (10.3)
Enterococcus faecium	1	8	7	13	1	0	15	1	22	1	11	0	2	82 (8.4)
**Gram-positive, other**	0	2	1	6	1	0	3	1	1	0	0	0	0	15 (1.5)
**Clostridioides**	0	0	3	1	0	0	1	8	0	0	0	0	0	13 (1.3)
**Gram-negative bacteria**	60	27	38	15	32	0	55	18	7	1	4	2	6	265 (27.1)
**Funghi**	10	5	0	3	25	1	15	4	0	0	0	0	3	66 (6.8)
**Vancomycin resistant enterococci**	0	0	1	1	0	0	1	0	0	0	0	0	0	3 (0.3)
**Other**	0	3	1	0	0	0	2	0	0	0	0	0	0	6 (0.6)

Data are presented as absolute (relative frequencies).

Table S2. Multivariate analysis of variance on the impact of BMI and RRT on vancomycin serum concentrations stratified by BMI groups.

BMI (kg m^{-2})	Total		18.5 – 29.9		30.0 – 34.9		35.0 – 39.9		> 40	
	RRT	No RRT	RRT	No RRT	RRT	No RRT	RRT	No RRT	RRT	No RRT
Days to target concentrations	3 (2; 4)	3 (2; 4)	3 (2; 4)	3 (2; 4)	3 (2; 4)	3 (2; 4)	3 (2; 4)	3 (2; 4)	3 (1; 4)	3 (1; 4)
Vancomycin therapy days	5 (3; 8)	5 (3; 8)	3 (5; 8)	3 (5; 8)	4 (6; 11)	4 (2; 7)	4 (3; 7)	3 (5; 11)	3 (2; 9)	5 (3; 9)
Therapy days < 25 mg L^{-1}	2 (1; 3)	2 (1; 3)	2 (1; 3)	2 (1; 3)	2 (1; 3)	2 (1; 3)	1 (1; 3)	2 (0; 4)	1 (0; 2)	2 (0; 4)
Therapy days in target range	1 (0; 2)	1 (0; 2)	2 (1; 3)	2 (1; 3)	2 (1; 3)	2 (1; 3)	1 (1; 3)	1 (1; 3)	1 (1; 2)	1 (1; 2)
Therapy days > 25 mg L^{-1}	1 (0; 2)	0 (0; 2)	1 (0; 2)	1 (0; 2)	1 (1; 3)	1 (0; 2)	1 (1; 2)	1 (0; 3)	1 (0; 4)	1 (0; 3)

All data are given as median (25th; 75th percentile).

Table S3. Multivariate Tests. Results of the multivariate analysis of variance on the impact of BMI, RRT and their interaction on vancomycin serum concentrations.

Effect	Test used	Value	F	df	Error df	Significance
Intercept	Pillai's Trace	0.622	185.410b	5.000	564.000	<0.001
BMI group	Pillai's Trace	0.018	0.675	15.000	1.698.000	0.811
RRT	Pillai's Trace	0.011	1.236b	5.000	564.000	0.291
BMI group * RRT	Pillai's Trace	0.023	0.862	15.000	1.698.000	0.608

Df: degrees of freedom; BMI: body mass index; RRT: renal replacement therapy

Table S4. Multivariate analysis. Tests of between-subject effects.

Source	Dependent Variable	Sum of Squares	df	Mean Square	F	Significance
**Corrected Model**	vanco_days	123.386	7	17.627	1.394	0.205
	days_to_target	11.176	7	1.597	0.413	0.894
	days_underdosed	18.035	7	2.576	0.717	0.657
	days_inrange	12.800	7	1.829	0.932	0.481
	days_overdoseed	44.609	7	6.373	1.941	0.061
**Intercept**	vanco_days	5.375.099	1	5.375.099	425.225	<0.001
	days_to_target	1.483.929	1	1.483.929	384.071	<0.001
	days_underdosed	677.594	1	677.594	188.632	<0.001
	days_inrange	622.351	1	622.351	317.051	<0.001
	days_overdoseed	457.604	1	457.604	139.348	<0.001
**BMI group**	vanco_days	3.447	3	1.149	0.091	0.965
	days_to_target	8.582	3	2.861	0.740	0.528
	days_underdosed	7.118	3	2.373	0.660	0.577
	days_inrange	8.865	3	2.955	1.505	0.212
	days_overdoseed	6.368	3	2.123	0.646	0.585
**RRT**	vanco_days	2.222	1	2.222	0.176	0.675
	days_to_target	0.662	1	0.662	0.171	0.679
	days_underdosed	5.100	1	5.100	1.420	0.234
	days_inrange	0.265	1	0.265	0.135	0.714
	days_overdoseed	12.495	1	12.495	3.805	0.052
**BMI group* RRT**	vanco_days	69.870	3	23.290	1.842	0.138
	days_to_target	1.022	3	0.341	0.088	0.967
	days_underdosed	3.350	3	1.117	0.311	0.818
	days_inrange	2.838	3	0.946	0.482	0.695
	days_overdoseed	21.387	3	7.129	2.171	0.090

df: degrees of freedom; BMI: body mass index; RRT: renal replacement therapy
